# Psocoptera of Canada

**DOI:** 10.3897/zookeys.819.27640

**Published:** 2019-01-24

**Authors:** Johannes E. Anonby

**Affiliations:** 1 Fylkesmannen i Sogn og Fjordane, Njøsavegen 2, NO-6863 Leikanger, Norway Fylkesmannen i Sogn og Fjordane Leikanger Norway

**Keywords:** bark lice, biodiversity assessment, Biota of Canada, book lice, Psocoptera

## Abstract

At present, 108 species of Psocoptera are known from Canada, an increase of 50% from the known fauna reported in 1979. Of these, 56 have been barcoded, representing 162 Barcode Identification Numbers (BINs). An additional 67 species are expected to occur in Canada but remain undiscovered or undescribed, meaning that only 62% of the fauna is currently documented.

Psocoptera, commonly known as bark lice and book lice, comprised about 4400 species known worldwide by the time the World catalogue was published, with 294 species in North America (Lienhard and Smithers 2002). By the end of 2015, the worldwide species number had increased to 5958 (C Lienhard pers. comm.). Mockford (2012) lists 397 species from North America south to the Tropic of Cancer.

Psocoptera are generally herbivores or detritivores, feeding on microflora and organic debris. Species associated with human dwellings, most belonging to the suborders Trogiomorpha and Troctomorpha, often feed on molds as well as dead insects. The outdoor-living species, mostly of the suborder Psocomorpha, may be classified as either bark-dwellers or leaf-dwellers, with associated differences in feeding habits (e.g., Mockford 1993, Lienhard 1998). Many domiciliary species are found outdoors if conditions are favourable. Since indoor species are readily spread by human commerce around the world, it may often be difficult to say where they originally came from, and whether they are native or introduced species or populations.

Although classified as an order for much of recent history, Yoshizawa and Lienhard (2010) showed that Phthiraptera, the true lice, have evolved within the Troctomorpha suborder of Psocoptera. Phthiraptera and the family Liposcelididae of Psocoptera are probably sister groups, or various lines of Phthiraptera may have budded off independently in the infraorder Nanopsocetae within Troctomorpha. To maintain monophyly, the former orders of Psocoptera and Phthiraptera are now placed in the order Psocodea. For practical reasons, however, since true lice and psocids have quite different ecology, and are studied by different methods and by different experts, Psocoptera is still typically treated as a group in the traditional way, but referred to as Psocodea: ’Psocoptera’. This practical approach is also adopted in the present work.

Since the last review of Canadian Psocoptera (Mockford 1979), there has been relatively little effort focused on this group in Canada, and Canada has never had an expert working on this group. However, fortunately there have been some advances in understanding the composition, distribution, identification, and biogeographical affinities of the Canadian fauna through the work of Edward L. Mockford (USA). His handbook for identification of North American Psocoptera is mainly based on studies in the USA (Mockford 1993). However, since species numbers of Psocoptera generally decrease rapidly with latitude, and there seems to be few species that are restricted to the boreal or northwards, the handbook covers Canadian species very well. The handbook divided the North American Psocoptera species into distinct distribution patterns, whose corresponding geographic areas extended into Canada to various degrees, as well as listed introduced species. The biogeographic analysis was further developed in Mockford (2012) who discussed modes of dispersal, which is relevant to understanding the Canadian fauna as part of the North American and worldwide fauna. The website http://Psocodea.SpeciesFile.org provides much information on Psocodea: ’Psocoptera’, although it is not very specific for the Canadian fauna.

Mockford (1979) reported 72 known species from Canada and estimated that an additional 31 species remained to be discovered or described from the country, thus predicting a total fauna of about 103 species. Lienhard (2016) found that the world catalogue by Lienhard and Smithers (2002) contained references to 83 species reported from Canada. However, the report of *Blastopsocussemistriatus* (Walsh) in Mockford (2002) had gone unnoticed since it was published too late to be included in the catalogue. The number of Canadian Psocoptera species known by 2016 was therefore 84.

I collected more than 4000 specimens of Psocoptera during two months in Canada in 1993, mainly from British Columbia and Ontario with a few samples from Alberta and Yukon. The material was identified by me, with more difficult cases confirmed or corrected by Edward Mockford. Although the records were not published, this collection added 12 additional described species to the national checklist, not counting an *Anomopsocus* sp. from the Montane Cordillera of British Columbia that may represent a new species.

The Barcode of Life Data System database (BOLD) (Ratnasingham and Hebert 2007) contains more than 14,000 DNA barcodes for specimens of Psocoptera from Canada. Among the 162 BINs represented by Canadian specimens, there are another 12 named species that represent new Canadian records of described species. Thus, the number of currently known species of Canadian Psocoptera is 108, an increase of 50% since 1979 (Table 1).

**Table 1.** Census of Psocoptera in Canada.

Taxon^1^	No. species reported in Mockford (1979)	No. species currently known from Canada^2^	No. BINs^3^ available for Canadian species	Est. no. undescribed or unrecorded species in Canada	General distribution by ecozone^4^	Information sources
**Suborder Trogiomorpha**						
Lepidopsocidae	2	3	3	1	Pacific Maritime, Mixedwood Plains, Boreal Shield, Atlantic Maritime	Lienhard 2016; BOLD^5^
Trogiidae	3	4 (2)	6	2	outdoor spp.: Pacific Maritime, Western Interior Basin, Mixedwood Plains, Atlantic Maritime; some domiciliary	Lienhard 2016; Anonby unpubl. data
Psyllipsocidae	0	2 (2)	1	0	domiciliary. Very few records.	Anonby unpubl. data; BOLD
**Suborder Troctomorpha**						
Liposcelididae	2	6 (1)	15	9	outdoor spp: Western Interior Basin, Boreal Plains, Mixedwood Plains, Boreal Shield, Atlantic Maritime; some domiciliary	Lienhard 1998, 2016; Anonby unpubl. data
**Suborder Psocomorpha**						
Epipsocidae	2	2	5	2	Montane Cordillera, Boreal Shield, Mixedwood Plains, Atlantic Maritime	Lienhard 2016
Caeciliusidae	13	14	19	8	probably all ecozones	Mockford 1999, Lienhard 2016; Anonby unpubl. data; BOLD
Stenopsocidae	0	1 (1)	2	1	Pacific Maritime, Western Interior Basin, Montane Cordillera, Mixedwood Plains, Atlantic Maritime	Lienhard 2016
Amphipsocidae	1	1	1	0	southern, but reaching boreal ecozones.	Mockford 1993, Lienhard 2016
Dasydemellidae ^6^	1	1	2	1	Boreal Shield and all ecozones south of boreal, except Prairies.	Mockford 1993, Lienhard 2016
Lachesillidae	11	16 (1)	27	13	probably all ecozones	Lienhard 2016; Anonby unpubl. data; BOLD
Ectopsocidae	2	5 (1)	6	1	Pacific Maritime, Mixedwood Plains, Atlantic Maritime	Lienhard 2016; Anonby unpubl. data; BOLD
Peripsocidae	5	6 (1)	7	2	Boreal Plains, Boreal Shield and all ecozones south of boreal	Lienhard 2016
Trichopsocidae	0	1 (1)	1	0	Pacific Maritime	BOLD
Philotarsidae	2	4	5	1	Pacific Maritime, Western Interior Basin, Montane Cordillera, Mixedwood Plains, Atlantic Maritime	Lienhard 2016; BOLD
Elipsocidae	3	8 (4)	11	5	Pacific Maritime, Western Interior Basin, Montane Cordillera, Mixedwood Plains, Atlantic Maritime	Lienhard 2016; BOLD
Mesopsocidae	2	3 (1)	6	2	probably all ecozones except Arctic	Lienhard 2016
Psocidae	22	29 (2)	41	18	probably all ecozones except Arctic	Lienhard 2016; Anonby unpubl. data; BOLD
Myopsocidae	1	2	3	1	Mixedwood Plains, Atlantic Maritime	Lienhard 2016
**Total**	**72**	**108 (17)**	**162**	**67**		

^1^Classification follows Lienhard and Smithers (2002) but infraorders are not indicated. ^2^Numbers in parentheses represent the number of non-native species (according to Mockford 1993) included in the total. ^3^Barcode Index Number, as defined in Ratnasingham and Hebert (2013). ^4^See figure 1 in Langor (2019) for a map of ecozones. ^5^Barcode of Life Data Systems database (www.boldsystems.org). ^6^Dasydemellidae was split off from Amphipsocidae by Mockford (1993).

Although a large number of Canadian specimens have been barcoded, there are still 52 recorded species that have not yet been sampled genetically. Furthermore, some of the barcoded Canadian material has not yet been identified to species, and in some cases identification is incorrect, so it is possible that once these identifications are confirmed or corrected, additional known Canadian species will have associated BINs. The estimates of yet-undocumented species in Canada were calculated based on available number of BINs not yet assigned to Canadian species and consideration of the number of species (21) present in adjacent states of the USA but still not found in Canada but likely to be there. Undocumented species will likely include unidentified described species and undescribed taxa (including cryptic species). Using an approach that recognizes the likelihood that not every BIN represents a unique species, that species may share BINs, and that not every species in Canada has been barcoded, it is conservatively estimated that another 67 species occur in Canada, representing 38% of the total anticipated fauna (Table 1).

In general, the Psocoptera fauna of Canada is not well sampled so that even modest inventory effort could result in new jurisdictional records. New species remain to be discovered and described in all ecozones. New taxa may sometimes be found in places where they are not expected, and where there are not many insects at all, such as in caves and other underground habitats. The Arctic ecozone should not be forgotten, even though the number of species may be very low. Odd-looking, winged Psocoptera have been found on barren rocks in mountains north of the tree line in Norway (J Anonby unpubl. data), so it is likely that sampling various habitats in the vast northern parts of Canada will reveal new species which are rare or absent farther south. Continued barcoding efforts will help identify cryptic species, elucidate intraspecific genetic diversity, and help detect rare and relict species that may require conservation measures as well as non-native species that may be threatening natural ecosystems. The distinction between native and introduced species may be particularly demanding in Psocoptera, given regular long-distance dispersal in many species.
